# Alpha-galactosylceramide as mRNA vaccine adjuvant modulates cellular immunity against tuberculosis and contributes to antigen-specific lung-associated T cells with a TRM-associated phenotype following prime-pull strategy

**DOI:** 10.3389/fimmu.2026.1929726

**Published:** 2026-10-05

**Authors:** Ilke Aernout, Thomas Ehouarne, Kevin Mwangi, Kia C. Ferrell, Margo De Velder, Janne Swinnen, Aaron Verplancke, Laura Van Moortel, Margaux De Meyer, Patrick Willems, Lorenzo Franceschini, Karine Breckpot, Stefaan C. De Smedt, Francis Impens, Caroline Demangel, Rein Verbeke, Ine Lentacker

**Affiliations:** 1 Laboratory of Physical Pharmacy and General Biochemistry, Department of Pharmaceutics, Ghent Research Group on Nanomedicines, Ghent University, Ghent, Belgium; 2 Immunobiology and Therapy Unit, Inserm U1224, Institut Pasteur, Université Paris Cité, Paris, France; 3 Laboratory of Pharmaceutical Biotechnology, Faculty of Pharmaceutical Sciences, Ghent University, Ghent, Belgium; 4 Department of Biomolecular Medicine, Ghent University, Ghent, Belgium; 5 Vlaams Instituut voor Biotechnologie (VIB) Center for Medical Biotechnology, VIB, Ghent, Belgium; 6 Translational Oncology Research Centre, Department of Biomedical Sciences, Vrije Universiteit Brussel (VUB), Brussels, Belgium

**Keywords:** adjuvants, mRNA vaccine, *Mycobacterium tuberculosis*, prime-pull strategy, T cells with TRM-associated phenotype

## Abstract

Tuberculosis (TB) remains the leading cause of death from a single infectious agent worldwide, highlighting the need for improved vaccines beyond the limited protection currently provided by the Bacillus Calmette-Guérin (BCG) vaccine. Here we investigated the immunological impact of incorporating alpha-Galactosylceramide (αGC) into mRNA lipid nanoparticles (mRNA-LNPs) as a vaccination strategy against *Mycobacterium tuberculosis* (Mtb), the pathogen causing TB. Using Mtb antigens ESAT-6 and Ag85B, we demonstrate that αGC-adjuvanted mRNA-LNPs induce controlled activation of invariant natural killer T (iNKT) cells, thereby reshaping vaccine-induced immunity. Specifically, αGC incorporation promoted the generation of CD44^+^CD69^+^CD62L^-^CD4^+^ T cells in the spleen and altered the CD4^+^ T -helper (T_H_) profile through the addition of modest T_H_2 and T_H_17 responses alongside robust T_H_1 immunity. Furthermore, antigen-specific CD8^+^ T-cell responses against ESAT-6 and Ag85B were observed exclusively in the genetically diverse CB6F1/J mouse model, highlighting the importance of host genetic background in evaluating mRNA vaccine immunogenicity after homologous vaccination strategies. Finally, we demonstrate that using a heterologous prime–pull strategy combining intramuscular mRNA vaccination with local antigen instillation increased the frequency of antigen-specific lung-associated CD4^+^ T cells displaying a tissue residency-associated phenotype, with additional contribution of αGC adjuvantation to this response. In summary, these findings provide novel insights into αGC adjuvant-mediated immune modulation of mRNA vaccines and support further testing of protective efficacy of this approach against TB.

## 1 Introduction

Tuberculosis (TB) caused by *Mycobacterium tuberculosis* (Mtb) remains the world’s leading cause of death from a single infectious agent. According to the latest World Health Organization (WHO) report, an estimated 10.7 million people developed TB in 2024, and a total of 1.23 million deaths were attributed to the disease (1). The global TB burden is further aggravated by co-infection with human immunodeficiency virus (HIV) and by the emergence of multidrug-resistant (MDR) strains. This underscores the urgent need to develop new tools and interventions that prevent transmission and/or effectively treat the disease (1, 2). Effective vaccination is widely recognized as a critical pillar in global TB elimination efforts. The only licensed vaccine, the live-attenuated *Mycobacterium bovis* strain Bacillus Calmette-Guérin (BCG), provides reliable protection in children against mostly meningeal and disseminated TB, but shows low and highly variable efficacy against pulmonary TB in adults (3–5). With the WHO’s ambitious goal of ending TB by 2035, substantial efforts have been directed toward the development of improved TB vaccine candidates, either to replace BCG or to boost its efficacy (6). As a result, 17 vaccine candidates are currently in clinical development (1, 7).

In 2020, the coronavirus disease 2019 (COVID-19) pandemic accelerated the development of mRNA vaccines, firmly establishing them as a promising next-generation vaccine platform. These vaccines demonstrated a favorable safety profile, and high efficacy in preventing SARS-CoV-2 infection and severe illness (8, 9). Given the growing interest in mRNA vaccine technologies, the rapid initiation of efforts to develop mRNA vaccines targeting Mtb was not surprising. mRNA vaccines composed of mRNA packaged in lipid nanoparticles (mRNA-LNPs) are particularly effective in inducing humoral immunity, while also eliciting robust systemic CD8^+^ T-cell responses alongside polyfunctional CD4^+^ T cells (10, 11). Moreover, mRNA vaccines offer great versatility because they can encode multiple antigens within a single mRNA vaccine (12). This feature is highly relevant in the context of TB, where multivalent vaccines are likely necessary to address antigenic variation among Mtb strains and to provide protection across different stages of infection (13). BioNTech has already advanced two multivalent clinical candidates, BNT164a1 and BNT164b1 (14), encoding eight Mtb antigens, which are currently being evaluated in a phase 1b/2a clinical trial in Mozambique and South-Africa (NCT05547464). In addition, several other studies have demonstrated protective efficacy of mRNA-based vaccines against Mtb in preclinical models (15–18).

For many years, IFN-γ secreting CD4^+^ T helper type 1 cells (T_H_1) and IL-17-secreting CD4^+^ T helper type 17 cells (T_H_17) have been considered the primary effector cell types in TB vaccine development, despite the lack of validated correlates of protection (19). Recent studies have additionally revealed major contributions of CD8^+^ T cells and unconventional T cells such as gamma delta T cells, iNKT cells, NK cells and Mucosal-associated invariant T (MAIT) cells, particularly during early control of Mtb infection (20, 21). Additionally, it is a challenge for vaccination strategies to maintain such responses, and thus to induce durable systemic memory and to ensure continuous replenishment of tissue-resident immune populations (22). Lung tissue resident memory T (T_RM_) cells and memory-associated cell types are therefore considered key mediators of early Mtb control and are proposed to play a central role in the induction of sterilizing immunity (22, 23). To promote the accumulation of T cells with a tissue residency-associated phenotype in the lung, various heterologous vaccination strategies have been explored. One promising approach is the prime-pull vaccination, in which a parenterally administered vaccine is followed by a mucosal antigen boost. This strategy has been investigated across multiple vaccine Mtb platforms, with some demonstrating enhanced protection (24, 25), while others reporting limited or no improvement on protective efficacy (26, 27).

In our previous work, we showed that incorporating the invariant natural killer T-cell (iNKT) ligand, alpha-galactosylceramide (αGC), into mRNA-LNPs can broaden both innate and adaptive immunity. This is mediated through the stimulation of iNKT cells, which reciprocally promote dendritic cell maturation and thereby induce more efficient T-cell priming, ultimately leading to improved cell-mediated responses and increased antibody titers (28, 29). In a prophylactic vaccination setting, we demonstrated that αGC-adjuvanted mRNA-LNPs conferred a drastic improvement in protection against *Listeria monocytogenes* in mice (29) and demonstrated better clinical outcomes in budgerigars vaccinated against another intracellular bacterium, *Chlamydia psittaci* (30). Others have reported the generation of liver-resident memory T cells by αGC-adjuvanted mRNA-lipoplexes in the context of malaria (31). In the context of TB, robust data supports the role of iNKT cells in protection. Combining αGC with BCG or a sublingual subunit vaccine containing the TB antigens Ag85B and ESAT-6, resulted in improved bacterial control (32, 33). Similarly, in a therapeutic context, co-administration of αGC with the antibiotic isoniazid has been shown to further enhance treatment efficacy in C3H/HeJ and C57BL/6 mice, underscoring the direct impact of these iNKT cells (34). Considering this evidence, we hypothesize that incorporating αGC into mRNA-LNP formulations against TB could enhance their utility as vaccine candidates.

In this study, we profiled immune responses elicited by mRNA-LNPs co-encapsulating the adjuvant αGC together with mRNA encoding the two clinically relevant Mtb antigens, ESAT-6 and Ag85B, in two different mice strains. These antigens were selected because they are among the most studied antigen targets in TB vaccine research (7). Their well-established immunogenicity profile also provides a broad framework for evaluating vaccine-induced immune responses. ESAT-6 (Rv3875, EsxA) is a key part of the ESX-1 secretion system and, due to its presence within the region of difference 1 (RD-1) locus, that is deleted in BCG, it is believed to play an important role in the attenuation of the BCG vaccine. Ag85B (Rv1886c), in contrast, is one of the most abundant proteins naturally secreted by Mtb and BCG during active growth (35). Multiple T-cell epitopes were identified for both antigens, and numerous studies have demonstrated strong immune responses to Ag85B and ESAT-6 through various vaccine platforms (36, 37). Moreover, some of these studies have reported partial protection against Mtb, supporting the selection of this antigen combination for our work (15, 38). We performed an in-depth immune characterization demonstrating that αGC-adjuvanted mRNA-LNPs encoding ESAT-6 and Ag85B induce a broader response with an increased frequence of CD44^+^CD69^+^CD62L^-^ CD4^+^ T cells to conventional mRNA-LNPs. In addition, we show that heterologous boosting with intranasally delivered protein antigens was associated with higher numbers of antigen-specific CD4^+^ T cells in the lungs and increased the frequency of cells displaying T_RM_-associated phenotypes.

## 2 Materials and methods

### 2.1 mRNA production

*In vitro* transcribed messenger RNA (mRNA) encoding truncated ovalbumin (OVA), ESAT-6 or Ag85B was prepared from the plasmid LMCT (pLMCT) after cloning each antigen sequence via Gibson assembly (NEB) using gBlocks (IDT) as described previously (39). Mycobacterial nucleotide sequences were obtained from the H37Rv Mtb reference strain accessed through Mycobrowser (40) and native signal sequences were removed when present. At the 5’ and 3’ ends, antigens were coupled to a sequence coding respectively for the signal peptide of the human LAMP1 protein and the transmembrane domain of human LAMP3 protein, to maximize both CD8^+^ and CD4^+^ T cell responses respectively (29).

The *in vitro* transcription reaction was performed starting from the linearized DNA template using a T7 enzyme mix containing T7 RNA polymerase (Thermo Fisher Scientific), RNase inhibitor (Promega), and inorganic pyro-phosphatase (Thermo Fisher Scientific). The reaction buffer mix included 10 mM Clean CAP AG reagent (TriLink Biotech),10 mM of dNTP (adenosine-, guanosine- and cytidine (Promega), and 10 mM of N1-methylpseudouridine (m1Ψ) (Trilink Biotech). After 2h incubation at 37 °C, 10 U DNase I exonuclease (Thermo Fisher Scientific) was added to the reaction mix and incubated for 15 min enabling the removal of residual dsDNA template. All enzymes added to the reaction were deactivated at 70 °C for 10 min. After digestion with DNase I, the mRNA was precipitated with LiCl and washed with 70% ethanol and subsequently dissolved in RNase-free water (Gibco). The purified mRNA was then subjected to standard quality controls, including spectrophotometric reading of optical density for yield determination and purity assessment (absorbance ratio at 260/280 nm) as well as evaluation of mRNA integrity (BioAnalyzer 2100, RNA 6000 chip).

### 2.2 mRNA- LNP production

LNPs consist of the ionizable lipid C12–200 and helper lipids cholesterol, DSPC (1,2-distearoyl-sn-glycero-3-phosphocholine) and DMG-PEG 2000 (1,2-dimyristoyl-rac-glycero-3-methoxypolyethylene glycol-2000). C12–200 and DSPC were purchased from Corden Pharma (Plankstadt, Germany) and all other lipids from Avanti Polar Lipids (Alabaster, USA). For the adjuvanted mRNA-LNPs, the additional lipid component, αGC (alpha-galactosylceramide) was added. Ionizable C12–200 LNPs containing mRNA were prepared as previously described (41). Briefly, lipid components (C12-200, DSPC, cholesterol, PEG-DMG) were dissolved in ethanol at a component molar ratio of ~50/10/38.5/1.5 with a final concentration of 5 or 10 mM total lipid. For the LNP formulations with αGC, 0.01 mol% of the total lipid amount was replaced by αGC, yielding a final αGC dose of 3.75 ng per injection of 2.5 µg mRNA-LNPs. mRNA (OVA or Ag85B and ESAT-6 in a 1:1 weight ratio) was dissolved in 25 mM sodium acetate buffer at pH 4.0 to obtain a final C12-200: mRNA weight ratio of 20:1. The organic and aqueous solutions were mixed through a T-junction mixer at a flow ratio of 1:3 (v:v) and a total flow rate of 20 mL min. The resulting suspension was dialyzed against a 200-500-fold volume 20 mM Tris buffer 9% sucrose at pH 7.4. The ionizable LNP formulations were subjected to a size and zeta potential quality control (**Supplementary Table 1**) using a Malvern Zetasizer nano-ZS (Malvern Instruments Ltd., Worcestershire, UK) by diluting it 50 times in HEPES 20mM PH7.4 or PBS PH7.4 (Gibco). The Quant-iT RiboGreen RNA Assay was used to determine mRNA encapsulation and concentration in lipid particle formulations according to manufacturer’s protocols (ThermoFisher, Waltham, MA). To release and detect the encapsulated mRNA content, mRNA particles were diluted in TE buffer containing 1% (v/v) Triton X-100 (Sigma) and incubated for 10 min at 37 °C, while the free (not-encapsulated) mRNA content was directly measured after particle dilution in TE buffer. mRNA-LNPs were concentrated or diluted to a concentration of 50 µg/mL using Amicon centrifugal tubes (100kDa MWCO) and stored at -80 °C until use.

### 2.3 Mice and vaccine administration

7-week-old female C57BL/6 or CB6F1/J mice were ordered from Envigo (The Netherlands) or Charles River laboratories (France), respectively. They were housed under controlled conditions (12h/12h light/dark cycle, temperature 21–24 °C, relative humidity 40–70%), where food and water were provided ad libitum.

Mice were vaccinated intramuscularly by injecting 50 µL of mRNA LNPs containing an mRNA dose of 0.5, 1,1.25, 2.5 or 5 µg, as defined in the figure subscriptions. Intranasal boosting was performed under light anesthesia by pipetting 30 µL (2.5 µg Ag85B- and 2.5 µg ESAT-6 protein in PBS) using a long pipet with microcapillary used for gel loading (VWR) in the right nostril. Proteins used for intranasal boosting were obtained from BEI-resources (Ag85B cat. NR-14857 and ESAT-6 protein cat. NR-49424). Weight of the animals was followed up at 0, 24, 48 and 72 hours post vaccination or following intranasal boost. Mice were given either prime i.m. vaccination only, prime-boost vaccination with 2 weeks interval or prime-boost with additional intranasal boost 1 week after i.m. mRNA-LNP boost vaccination. After 24, 72 hours, 2 weeks or 1 week post last injection, mice were sacrificed (see details in figures). Blood was collected from the central vein in heparin coated tubes (La Microvette^®^500, SARSTEDT) for analysis of sera. After perfusion of the lungs with 10 mL PBS, organs (spleen, inguinal lymph node and/or lungs) were harvested.

### 2.4 Sample collection and processing

Spleens were gently dissociated by pushing them through 40-μm cell strainers (Corning incorporated, Corning, US). Lymph nodes were enzymatically digested by incubating them twice for 10 min in RMPI 1640 (Thermofisher Scientific) containing 10 U/ml DNAse (Roche) and 0.02 mg/mL liberase (Roche). Lungs were processed according to the manufacturer’s protocol using the lung dissociation kit and OctoDissociator (Miltenyi Biotec, Germany). After single cell suspensions were obtained, a red blood cell lysis step was performed using a red blood cell lysis buffer (BioLegend, San Diego, CA, USA).

### 2.5 Flow cytometry of unstimulated cells

Unstimulated single-cell suspensions from spleen, draining lymph node or lungs were first incubated with a fixable viability dye “Zombie aqua” (Biolegend or Invitrogen™) and Fc Block (CD16/32, Trustain FcX) for 20 min at RT in PBS to prevent nonspecific Fc receptor binding. Subsequently, cells were stained with tetramers kindly provided by the National Institutes of Health (NIH) Tetramer Core Facility. Antigen-specific CD8^+^ or CD4^+^ T cells were stained with H-2Kb/SIINFEKL-BV421 (OVA tetramer) for 30 min at 4 °C in FACS or with I-A/QQWNFAGIEAAASA-PE (ESAT-6 tetramer) and I-A/FQDAYNAAGGHNAVF-BV421 (Ag85B tetramer) for 1 hour at 37 °C in RPMI 10% FBS (Thermofisher Scientific). iNKT cells were stained with mCD1d PBS-57 PE tetramer (iNKT-cell tetramer) for 30 min at 4 °C in FACS. Additional surface staining was performed using of combination of the following antibodies: TCRβ-APC Efluor780, PD1-FITC, CD8a-APC Fire750, TCRβ-APC, CD4-APC Fire 750, CD44-PerCP, KLRG1-BV605, CD69-FITC, CD62L-PE Vio770 for 30 min at 4 °C in FACS.

### 2.6 Flow cytometry staining of re-stimulated antigen-specific T cells

For re-stimulation of T cells, 5x 10^6^ splenocytes or lung cells were co-cultured with overlapping peptide pools covering the full protein sequence or with full-length proteins. For peptide-pool re-stimulation, 11AA overlapping 15-mer peptide pools spanning the entire sequence of ESAT-6 (UniProtKB Acc. no. P9WNK7) and Ag85B (UniProtKB Acc. no. P9WQP0) (Peptivator) were purchased from Miltenyi Biotec (Germany). Splenocytes were cultured with 2 µg/mL peptide pool in RPMI supplemented with 10% FBS (Thermofisher Scientific) and 2µg/mL Protein transport inhibitor cocktail (PTIC) containing Brefeldin A and Monensin (eBioscience) for 5 hours at 37 °C 5% CO_2_. After stimulation, cells were washed and processed for flow cytometry. For full length protein restimulation, T cells were co-cultured with 10 µg/mL ESAT-6 protein (cat. NR-49424) or Ag85B (cat. NR-14857) derived from BEI-resources in RMPI 10% FBS for 24 hours at 37 °C 5% CO_2_ after which the supernatant was collected for multiplex analysis. Re-stimulated splenocytes or lung cells were stained with fixable viability dye “zombie aqua” (Biolegend or Invitrogen™), and Fc block (CD16/32) for 20 min at RT in PBS to prevent non-specific Fc receptor binding to FcR (Trustain FcX). Subsequently cells were surface stained with CD8a-APC Fire 750, CD4-FITC, CD8a-APC, CD4-BV650, CD44-PerCP, KLRG1-BV605, PD-1-APC Fire 750, CD69-FITC and CD62L-PE Vio770 for 30 min at 4 °C in FACS. Additional staining with CXCR3 BV421 was performed for 1 hour at RT. Cells were then fixed and permeabilized with the intracellular Fixation & Permeabilization Buffer Set from eBioscience (Thermofisher Scientific). Intracellular cytokine staining was performed using IFN-γ -PE and/or TNF-α-BV421, IL-4-BV605, IL-17A-PerCP, IL-2-APC by incubation of the cells in permeabilization buffer for 30 min at RT.

After several washing steps, samples were acquired on a MACSQuant Analyzer 16 (Miltenyi Biotec, Germany) and analyzed using FlowJO™ software version 10.8.1. (BD biosciences). All antibodies were purchased from Biolegend, except for CD62L PeVio770 (Miltenyi Biotec) and TCRβ-APC Efluor780 (Invitrogen). More detailed information is provided in **Supplementary Table 4**). Compensation for spectral overlap was performed using UltraComp eBeads™ (Thermofisher Scientific) stained individually with each fluorochrome-conjugated antibody. Complete flow cytometric panels can be found in supplementary information.

### 2.7 Measurement of antibody responses using ELISA and cytokines using multiplex assay

Whole blood was collected in lithium-heparin coated tubes, centrifuged at 14,000 rpm for 5 min to separate plasma, after which samples were stored at -20 °C. The samples were diluted 1: 100 before anti-OVA IgG1 and IgG2c titers or anti-ESAT-6 and anti-Ag85 total IgG titers were quantified with commercial mouse antibody assay kits (Chondrex, Woodinville, USA and Alpha Diagnostic Intl. Inc, Texas, USA respectively), according to the manufacturer’s instructions. Cytokines were measured in the supernatant of re-stimulated splenocytes with full length protein for 24 hours after a two-fold dilution. A panel of cytokines, including IFN-γ, IL-5, TNF-α, IL-2, IL-6, IL-4, IL-10, IL-9, IL-17A, IL-17F, IL-22 and IL-13 was quantified using a multiplex assay (LEGENDplex™ Mouse Th cytokine 12- plex panel, Biolegend) and analyzed with the LEGENDplex™ Data Analysis Software (Biolegend).

### 2.8 Statistical analysis

All statistical analyses were performed in GraphPad Prism Version 8.0.1.(Boston, MA). Where applicable, one-way ANOVA followed by Tukey’s test for multiple comparisons, or two-way ANOVA followed by Tukey’s range test as *post-hoc* analysis was performed. Data is expressed as mean ± SD. *p < 0.05, **p < 0.01, ***p < 0.001 and ****p< 0.0001 were considered significant.

## 3 Results

### 3.1 αGC-adjuvanted mRNA-LNPs activate iNKT cells and antigen-specific CD8^+^ T cells in a dose-dependent manner

We first defined a dose range in which αGC-adjuvanted mRNA-LNPs elicited maximal immune activation while minimizing reactogenicity. To this end, mRNA‑LNPs encoding the model antigen ovalbumin (OVA) were formulated with or without αGC and delivered at escalating doses (**Figures 1a, b**). Physicochemical characterization confirmed that co-encapsulation of the αGC adjuvant did not alter particle size, surface charge or mRNA encapsulation efficiency (**Supplementary Table 1**). C57BL/6 mice were then vaccinated intramuscularly with non-adjuvanted- mRNA-LNPs (at a 5µg mRNA dose) or increasing doses αGC-adjuvanted mRNA-LNPs (at a 0.5, 1, 2.5 or 5 µg mRNA dose; corresponding to 1.52, 3.04, 7.6 or 15.2 ng αGC, respectively) (**Figure 1b**). iNKT-cell responses were quantified 72 hours later in draining lymph nodes (dLN) and the spleen, defining iNKT cells as TCRβ^+^ CD1d tetramer^+^ cells (**Figures 1c, d**). Overall, iNKT frequencies remained comparable between adjuvanted and non-adjuvanted vaccines across doses, yet a modest trend toward increased iNKT migration and/or proliferation in the dLN was observed at higher αGC-adjuvanted mRNA-LNP doses (2.5 µg and 5µg). In contrast, expression of the activation marker, programmed cell death protein-1 (PD-1) on iNKT cells showed a clear dose-dependent increase in both tissues (**Figures 1e–g**), consistent with our earlier observations (29, 42).

**Figure 1:**
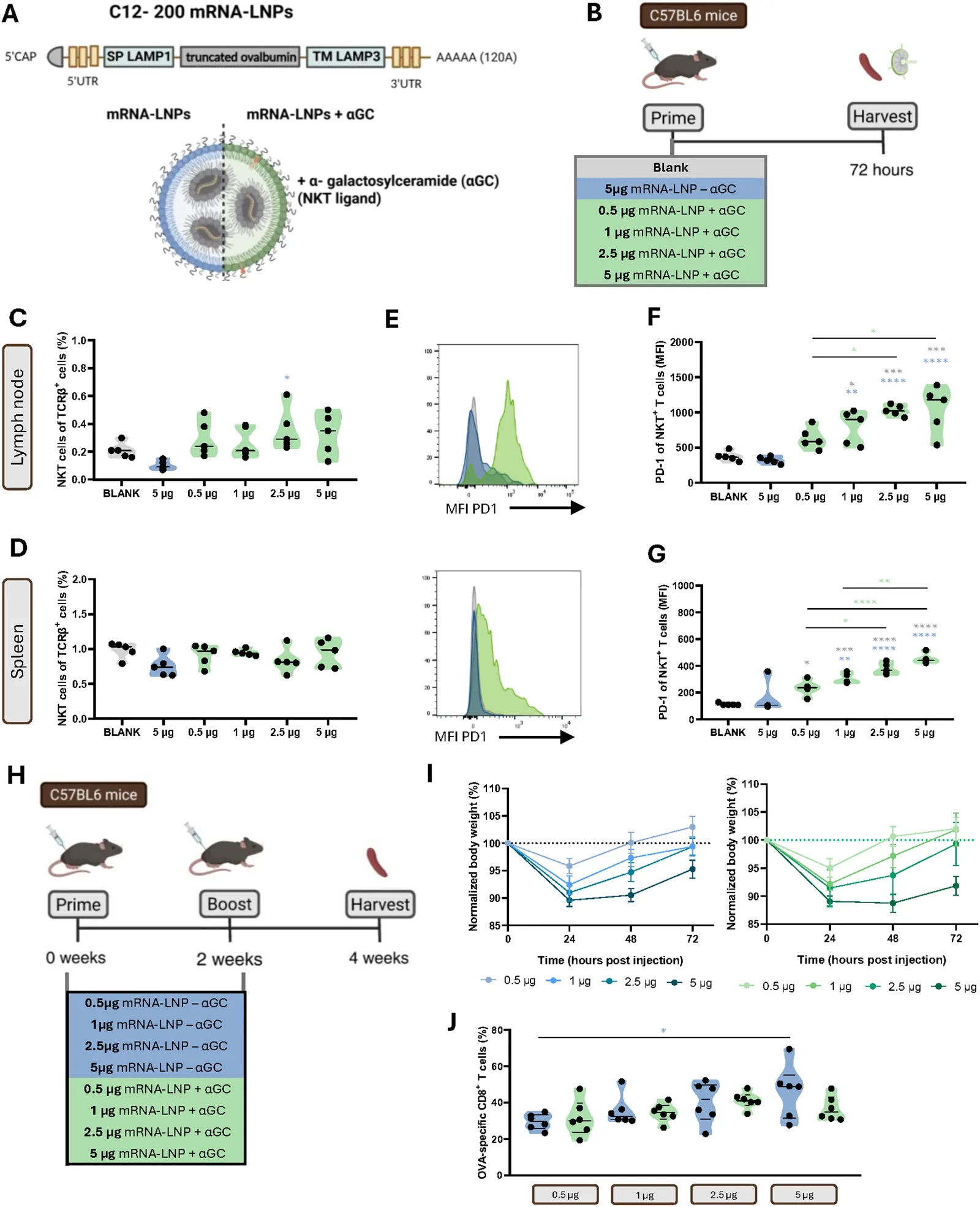
αGC in mRNA-LNPs activates iNKT cells and primes antigen-specific CD8^+^ T cells in a dose-dependent manner **(A)** Schematic representation of C12–200 mRNA-LNPs encapsulating m1Ψ-modified truncated ovalbumin (tOVA) mRNA encoding tOVA flanked by the LAMP1 signal peptide (SP) and LAMP3 trafficking motifs (TM) formulated as mRNA-LNPS without iNKT agonist, αGC (blue) or with (green). **(B)** C57BL/6 mice received a single intramuscular injection with various doses of the αGC-adjuvanted LNPs (0.5,1,2 and 5µg/mice), 5 µg of identical unadjuvanted mRNA-LNPs or Tris buffer. iNKT cell frequencies and activation status were assessed 72h later by flow cytometry. Frequency of iNKT cells in **(C)** draining lymph node (dLN) and **(D)** spleen. **(E)** Histograms of programmed cell death protein 1 (PD-1) expression (MFI) on iNKT cells as a measure for iNKT activation in dLN and spleen comparing unvaccinated mice (grey) with mice vaccinated with 5µg mRNA-LNP (blue) and 5µg αGC-adjuvanted mRNA-LNPs (green). **(F, G)** MFI of PD-1 expression on iNKT cells in dLN and spleen of mice vaccinated with 5µg of unadjuvanted mRNA-LNPs (blue) and increasing doses of αGC-adjuvanted mRNA-LNPs (green). **(H)** C57BL/6 mice received a prime and boost intramuscular injection with increasing doses (0.5,1,2.5 or 5µg/mice) of unadjuvanted (blue) or αGC-adjuvanted mRNA-LNPs (green) with a 2-week interval. Two weeks post boost the spleen was collected and analyzed with flow cytometry. (for statistic information see **Supplementary Table 2**) **(I)** Weights were monitored every 24 hours for a period of 72 hours after prime injection. **(J)** Frequency of antigen specific CD8^+^ T cells (H-2Kb/SIINFEKL tetramer) in the spleen. Each individual point represents one animal. Results given as mean ± SD, n=5 per group. P values were determined by a one-way **(C, D, F, G, J)** or two-way **(I)** ANOVA followed by Tukey’s *post-hoc* test. See **Supplementary Figures 1** and **2** for flow gating strategy.

To assess tolerability and adaptive immune responses, mice received two intramuscular injections of mRNA-LNPs with or without αGC at doses of 0.5, 1, 2.5 or 5 µg mRNA (**Figure 1h**). Body weight monitoring revealed a clear dose-dependent weight reduction following vaccination, reaching up to 10% relative weight loss at the highest dose (5 µg mRNA). No significant additional effect on body weight loss was observed following adjuvant incorporation. However, vaccinated mice receiving aGC showed a slightly slower recovery of body weight within 72 hours. (**Figure 1i**; **Supplementary Table 2**). Two weeks after boost immunization, OVA-specific CD8^+^ T-cell responses were evaluated in the spleen (**Figure 1j**). No statistically significant differences were observed between the different doses or between αGC-adjuvanted and non-adjuvanted formulations, except for the mice receiving 5 µg compared to 0.5µg of the unadjuvanted mRNA-LNPs, but a dose-response trend was apparent with the CD8^+^ T-cell response plateauing at the 2.5 µg dose. Notably, the CD8^+^ T-cell response in mice vaccinated with αGC-adjuvanted mRNA-LNPs displayed reduced inter-individual variability compared with the non-adjuvanted controls. OVA-specific IgG1 and IgG2c antibody levels remained below detection limits (data not shown), which is consistent with the design of the mRNA-encoded protein, which is not secreted and contains trafficking motifs of the human LAMP protein (**Supplementary Table 3**), optimized to predominantly elicit cell-mediated immunity rather than humoral responses (13, 29). For that reason, assessment of adjuvant-mediated effects on antigen-specific humoral immunity was beyond the scope of the current construct design, and the present study focused exclusively on cellular immune responses Based on these results, a dose of 2.5 µg mRNA-LNPs (for both with and without adjuvant) was selected for the subsequent experiments.

### 3.2 Inclusion of αGC in mRNA-LNPs balances CD4^+^ T_H_1/T_H_2 responses and enhances the generation of CD44^+^ CD69^+^CD62L^-^ CD4^+^ T cells

Next, we evaluated adaptive immunity induced by mRNA vaccination in C57BL/6 against the Mtb antigens ESAT-6 and Ag85B. The mRNA constructs were designed to enhance cell-mediated immunity by incorporating a signal peptide from human LAMP 1 (SP) to direct it into the secretory pathway, followed by a LAMP-3 derived lysosomal targeting sequence to promote endosomal routing and MHC presentation (**Supplementary Table 3**). These trafficking signals target expressed antigens to endo-lysosomal compartments containing both MHC class I and MHC class II molecules, thereby enhancing antigen presentation to CD8^+^ and CD4^+^ T cells (29, 43). Both Mtb antigen mRNAs were mixed at a 1:1 weight ratio and formulated into either unadjuvanted or αGC-adjuvanted mRNA-LNPs (**Figure 2a**). Two weeks after the booster dose, dLNs and spleens were collected for flow cytometric analysis of antigen-specific T cells using I-Ab tetramers against ESAT-6 (4-17) and Ag85B (280-294) epitopes and primary activation marker CD44^+^ (**Figure 2b**). Antigen-specific CD44^+^ CD4^+^ T cells were clearly identified in dLN from mice vaccinated with the unadjuvanted mRNA-LNP, whereas a smaller proportion of activated, antigen-specific T cells were detected in mice receiving the mRNA-LNPs with αGC (**Figure 2c**). In the spleen, ESAT-6-specific CD4^+^T cells were induced by both vaccine formulations to a similar degree (**Figure 2d**), but no significance was found for the one against Ag85B. T-cell memory markers, CD69 and CD62L, were also included to assess T cell activation and memory-associated phenotypes (44). In the spleen, the proportion of CD44^+^CD69^+^CD62L^-^ CD4^+^ T cells, was significantly enhanced by αGC inclusion for both ESAT-6 and Ag85B. Additional KLRG1 staining indicated that the majority of these cells were negative for KLRG1 (**Supplementary Figure 6**) (45).This increase was not observed in the dLN (data not shown). The αGC-induced upregulation of CD69 together with the concomitant downregulation of CD62L expression is consistent with the acquisition of an antigen-experienced phenotype. However, while these markers are indicative of activation and differentiation, they are not sufficient on their own to conclusively demonstrate commitment to an effector-memory phenotype.

**Figure 2:**
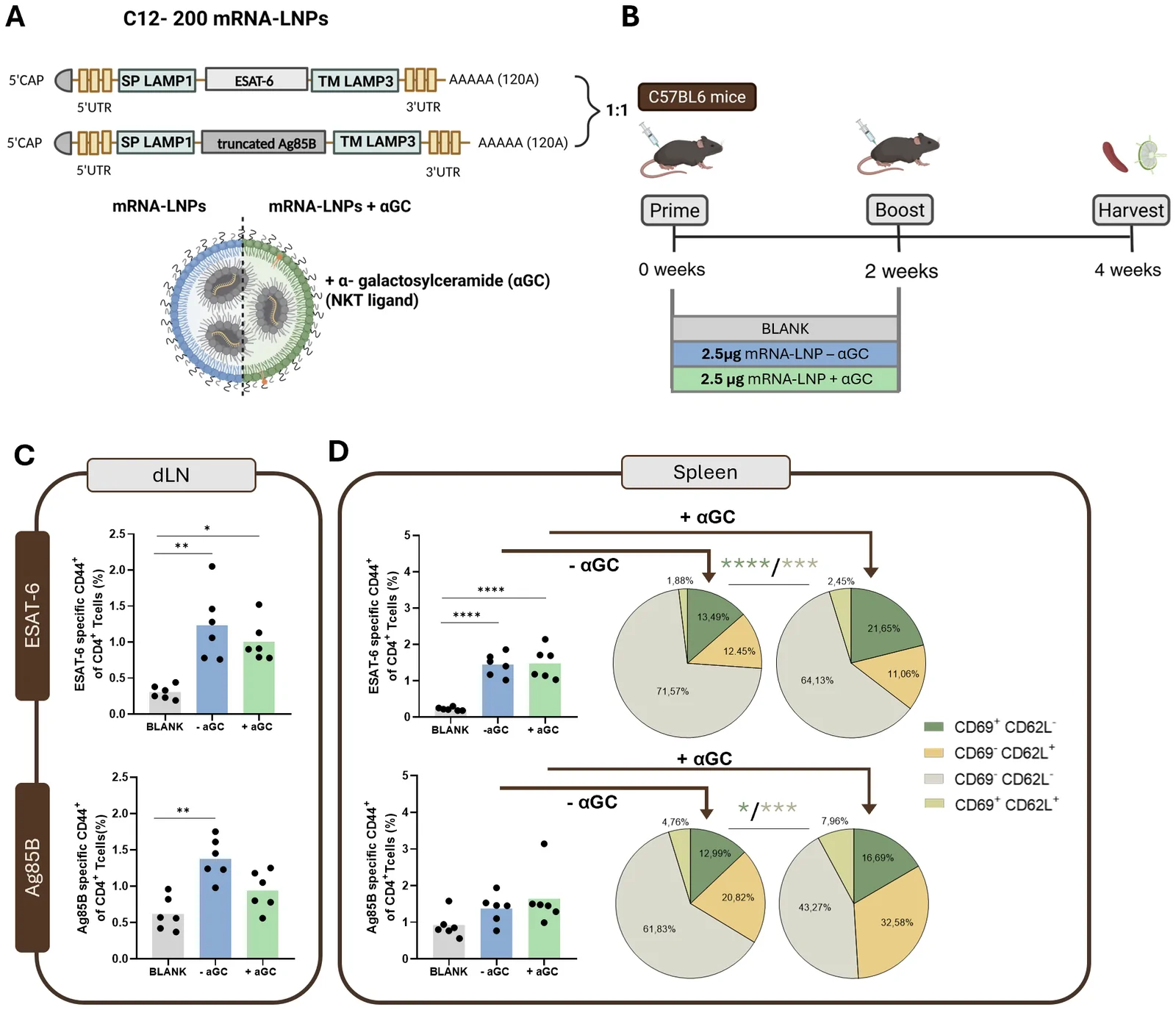
mRNA-LNPs and αGC-adjuvanted mRNA-LNPs induce Mtb antigen specific (memory) T cells after prime-boost vaccination in draining lymph node and spleen **(A)** Construct design of mRNA sequences encoding full length Mtb antigens ESAT-6 and truncated Ag85B flanked by sig-lamp signaling elements. Both mRNAs were co-formulated in a (1:1 weight ratio) in C12–200 LNPs (blue) or αGC-adjuvanted C12-200 LNPs (green), **(B)** Female C57BL/6 mice were vaccinated with 2.5 µg of the vaccines using a prime-boost regimen with a two-week interval. Two weeks after the boost vaccination draining lymph node (dLN) and spleen were collected for flow cytometry analysis. Antigen specific T cells were detected using ESAT-6 and Ag85B-specific I-Ab tetramers in **(C)** dLN and **(D)** spleen with frequencies of CD69_+_ CD62L_-_, CD69_-_ CD62L_+_, CD69_-_ CD62L_-_, CD69_+_ CD62L_+_ subsets in spleen subsets in spleen. Each individual point represents one animal, with bars or diagrams indicating mean, n=6 per group. P values were determined by a one-way ANOVA followed by Tukey’s *post-hoc* test. See **Supplementary Figure 3** for gating strategy.

By peptide-pool restimulation, we further characterized the polarization of the antigen specific CD4^+^ T-cell response in the spleen. Robust cytokine-producing CD4^+^ T cells were detected for both antigens, showing similar levels of CD4^+^ T cells producing T_H_1 cytokine levels IFN-γ and TNF-α for the two LNP formulations, except for a higher IFN-γ production following Ag85B restimulation in mice vaccinated with the αGC-adjuvanted mRNA-LNPs (**Figure 3a**). Strikingly, lower levels of IL-2 producing CD4^+^ T cells were detected in the presence of αGC. Consequently, the induction of polyfunctional T_H_1 cells (IFN-γ, TNF-α and IL-2 positive cells) was higher after vaccination with unadjuvanted mRNA-LNPs (**Figure 3b**). Intracellular staining for IFN-γ and TNF-α revealed no detectable CD8^+^ T-cell responses to either of the antigens (data not shown). While several MHC-I epitopes have been described in the literature for both antigens (36, 37, 46), it remains unclear whether H-2^b^ restricted epitopes, corresponding to the MHC-I haplotype expressed by C57BL/6 mice strains, are present, as previous studies often relied on different vaccine platforms and antigen combinations.

**Figure 3:**
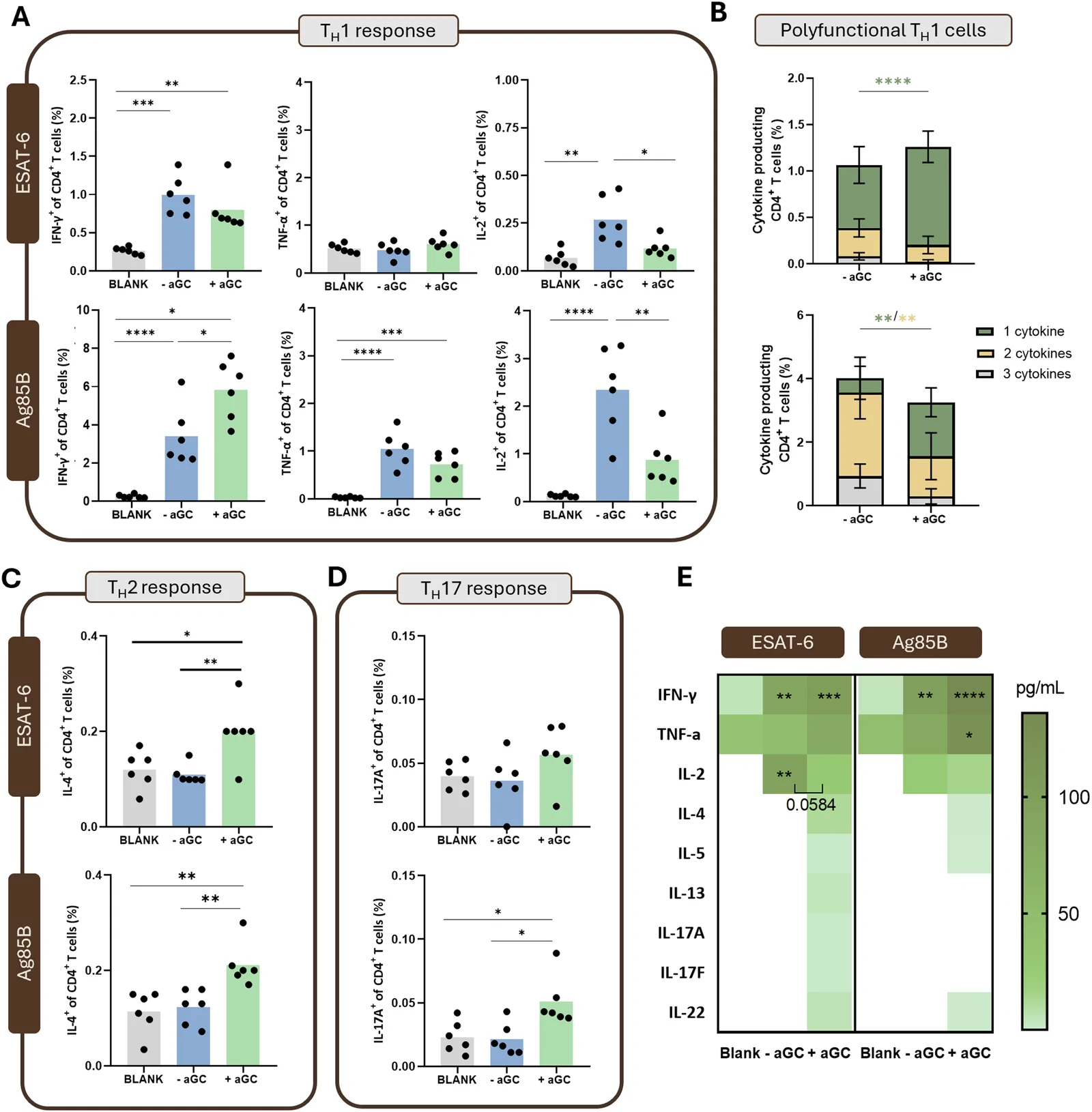
Impact of αGC inclusion on systemic CD4^+^ T_H_ responses after vaccination with mRNA-LNPs encoding Mtb antigens ESAT-6 and Ag85B. Female C57BL/6 mice were vaccinated with 2.5 µg mRNA-LNPs with or without the adjuvant αGC encoding full length Mtb antigens ESAT-6 and Ag85B in a prime boost regimen with a 2-week interval. Analysis of T_H_ responses after re-stimulation of splenocytes with ESAT-6 and Ag85B peptide pools: **(A)** Intracellular cytokine staining to detect T_H_1 CD4^+^ T cells (IFN-γ, TNF-α and IL-2); **(B)** polyfunctional T_H_1cells; **(C)** T_H_2 cytokine (IL-4) and **(D)** T_H_17 responses (IL-17A). **(E)** Cytokine levels in the supernatant of splenocytes restimulated with full length proteins ESAT-6 and Ag85B. Boolean gating was applied to analyze the co-expression of the polyfunctional cytokine producing CD4^+^ T cells (Y-axis range: 0–140 pg/mL). Each individual point represents one animal, with bars indicating mean or heatmap, n= 6 per group. P values were determined by one-way (or a two-way in case of polyfunctional T cells and **(E)** ANOVA followed by Tukey’s *post-hoc* test. Reported P values were corrected for multiple testing across all comparisons within the full grid using the Tukey adjustment. See **Supplementary Figure 4** for gating strategy.

Restimulation with the Ag85B overlapping peptide elicited stronger overall responses than ESAT-6, despite the lower molar dose of mRNA encoding Ag85B in the vaccine formulation as a weight-based mRNA ratio was used in the LNP formulations (**Figure 2b**). Interestingly, T_H_2 cytokines were detected in mice vaccinated with αGC-adjuvanted mRNA-LNPs (**Figure 3c**), indicating that the inclusion of αGC altered the polarization of the vaccine-induced CD4^+^ T-cell response, while maintaining substantial T_H_1 responses. Finally, αGC‑adjuvanted mRNA‑LNPs elicited a detectable T_H_17 response against Ag85B (**Figure 3d**), with a significantly increased proportion of IL-17A and IFN-γ double positive CD4^+^ T-cells (**Supplementary Figure 7**).

To further validate these findings, splenocytes from the same mice were stimulated in parallel with full-length antigen proteins (**Figure 3e**). Cytokine levels measured in the supernatants confirmed the trends observed following peptide-pool re-stimulation. In these experiments, we assessed a broader T_H_2 cytokine panel, which revealed IL-5 secretion in response to both antigens, as well as IL13 secretion specifically in response to ESAT\‑6, when vaccinated with the adjuvanted mRNA-LNPs. These results further support that αGC promotes a more balanced T_H_1/T_H_2 profile. Upon ESAT-6 restimulation, we were able to detect low levels of IL-17, supporting the previous observation of T_H_17 CD4^+^ T‑cell responses. IFN-γ and TNF-α levels were slightly elevated in mice vaccinated with αGC-adjuvanted mRNA-LNPs. In contrast, intracellular cytokine staining of CD4^+^ T cells did not reveal increased overall median fluorescence intensity signals for these cytokines. Cytokine levels measured in the restimulation assay reflect total production and are not indicative of per-cell cytokine level. This discrepancy may thus reflect the contribution of other innate immune cells, such as γδ T cells, to cytokine production upon protein restimulation, as previously reported (47, 48). In addition, lower levels of IL-2 detected in both the peptide and protein restimulation experiments may also be explained by usage of IL-2 by these unconventional T cells (49). Finally, it should be noted that the relatively high background signal detected in samples of unvaccinated mice can be linked to the inherent immune stimulation for ESAT-6, as reported before (50). ESAT-6 or Ag85B specific anti-IgG antibody levels remained below the detection limit.

### 3.3 Hybrid CB6F1 mice display both antigen-specific CD4^+^ and CD8^+^ T-cell responses and confirm αGC-associated T_H_2 cytokine production following mRNA-LNP vaccination

In our previous work, we demonstrated that αGC inclusion can strengthen CD8^+^ T-cell responses (28, 29). However, we were unable to detect ESAT-6 or Ag85B specific CD8^+^ T-cell responses in the C57BL/6 model. Interestingly, several H-2^d^ MHC-I haplotype-specific epitopes have been reported in literature for Ag85B, which are expressed in the BALB/c mouse strain (18, 51). Therefore, we conducted analogous experiments in the hybrid CB6F1 mouse strain, which have both the H-2^b^ and H-2^d^ MHC-I haplotypes (**Figure 4a**). As different mouse strains are known to show distinct T_H_1/T_H_2 immune biases, with C57BL/6 mice being generally T_H_1 dominant while BALB/c mice are T_H_2 dominant, we also sought to determine the impact of genetic background on vaccine-induced CD4^+^ T-cell responses. In addition, because our αGC-adjuvanted mRNA vaccine induced a population of T cells with high expression of CD69 in the spleen (**Figure 2d**), we investigated whether a similar phenotype could also be observed in the CB6F1 hybrid mouse model. Since upregulation of CD69 and downregulation of CD62L have been described in a specific circulating CD8^+^ T-cell population capable of giving rise to tissue-resident memory T cells (T_RM_), referred to as T_RM_ precursors (52), we asked whether the vaccine-induced CD44^+^CD69^+^CD62L^−^KLRG1^−^ CD4^+^ T-cell population observed in the spleen would be associated with increased frequencies of antigen-specific CD4^+^ T cells expressing T_RM_-associated markers in the lungs following a prime-pull strategy. To address this question, we employed a prime-pull vaccination strategy; CB6F1 mice received an intramuscular prime-boost vaccination of either unadjuvanted or αGC-adjuvanted mRNA LNPs encoding both ESAT-6 and Ag85B. One week after completion of the mRNA‑LNP prime–boost regimen, half of the mice received an intranasal instillation of a mixture containing full‑length ESAT‑6 and Ag85B proteins as a pull stimulus to promote the generation of antigen‑specific T cells in the lung (**Figure 4a**). As direct pulmonary administration of LNPs poses additional challenges related to delivery efficiency and safety, the preferred way to deliver the pull stimulus intranasally was using protein antigens (53, 54).

**Figure 4:**
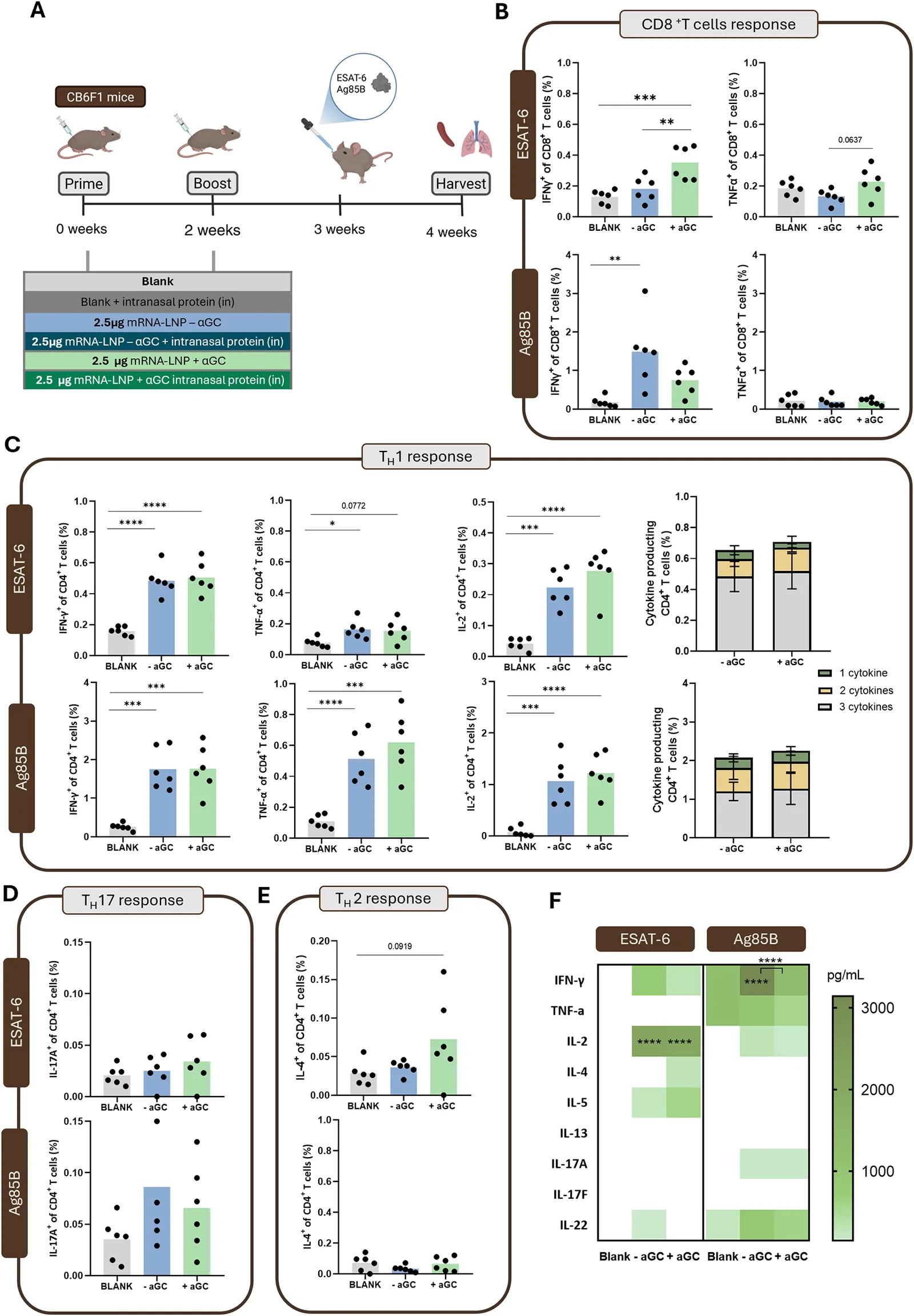
Impact of αGC inclusion on CD4^+^ and CD8^+^ T-cell responses in the spleen after vaccination with mRNA-LNPs encoding Mtb antigens ESAT-6 and Ag85B in CB6F1 mice: **(A)** CB6F1 mice were immunized intramuscularly with a prime-boost dose (two-week interval) of m1Ψ-modified mRNA encoding sig-Ag85B-LAMP3 and sig-ESAT-6-LAMP3 (1:1 weight ratio) mRNA formulated in C12–200 mRNA LNPs alone (2.5 μg mRNA/mouse) or co-formulated with αGC adjuvant (n = 6) and additionally boosted at week 3 with intranasal administration of the full length proteins Ag85B and ESAT-6 (1:1 weight ratio). After re-stimulation of splenocytes from non-pulled mice with ESAT-6 and Ag85B peptide pools separately antigen-specific T cell responses were analyzed **(B)** IFN-γ and TNF-α production by CD8^+^ T cells. **(C)** T_H_1 cytokine (IFN-γ, TNF-α and IL-2)-producing CD4^+^ T cells and polyfunctional T_H_1 cells, **(D)** T_H_17 (IL-17A) and **(E)** T_H_2 (IL-4) CD4^+^ T cells. **(F)** Confirmation of cytokine production via re-stimulation of splenocytes with 10 µg/mL full length proteins (ESAT-6 and Ag85B) and analysis of supernatant after 24 hours of restimulation (Y-axis range: 0–3200 pg/mL). Boolean gating was applied to analyze the co-expression of the polyfunctional cytokine producing CD4^+^ T cells. Each individual point represents one animal, with bars indicating mean (± SD for polyfunctional T_H_1 cells) or heatmap, n= 6 per group. P values were determined by one-way (or a two-way in case of polyfunctional T cells and **(G)** ANOVA followed by Tukey’s *post-hoc* test. Reported P values were corrected for multiple testing across all comparisons within the full grid using the Tukey adjustment. See **Supplementary Figure 4** for gating strategy.

First, we were indeed able to detect ESAT-6 and Ag85B specific CD8^+^ T cells in this hybrid mouse strain. Surprisingly, the impact of αGC inclusion on these responses were variable and depended on the antigen, with increased CD8^+^ T cell responses for ESAT-6 when αGC was included, but a trend toward reduced response in formulations containing Ag85B and adjuvant (**Figure 4b**).

Next, our results showed that αGC inclusion had no detectable impact on T_H_1 or T_H_17 responses in the hybrid CB6F1 model, both at the single-cytokine and polyfunctional level. This contrasts with the observations in C57BL/6 mice, which displayed clear differences in T_H_1- and T_H_17-associated intracellular cytokine levels (IFN-γ, TNF-α, IL-2 and IL-17, respectively) in response to the adjuvant (**Figures 4c, d**, **3a, d**). These findings suggest that genetic background influences responsiveness to vaccine-mediated immune modulation. Similar observations have been reported in the context of a natural Mtb infection where C57BL/6 mice mount stronger T_H_1 and T_H_17 responses compared with BALB/c mice (55). However, we could confirm that αGC inclusion resulted in IL-4 producing CD4^+^ T_H_2 cells specific for ESAT-6 (**Figure 4e**). Cytokine production following whole antigen re-stimulation of splenocytes supported these findings, with increased absolute cytokine concentrations also suggesting a more pronounced T-cell activation in the hybrid mice compared to C57BL/6 mice (**Figures s 3e**, **4f**). This was particularly demonstrated by the high IFN-γ levels measured after restimulation of splenocytes with the Ag85B antigen.

### 3.4 αGC inclusion and local protein boosting increase frequencies of lung-associated antigen-specific CD4^+^ T cells displaying a T_RM_-associated phenotype

Tetramer staining for both antigens revealed that inclusion of αGC in the mRNA-LNP formulation significantly increased the frequency of Ag85B-specific CD4^+^ T cells in the spleen and elicited a similar, albeit non-significant, trend for ESAT-6-specific CD4^+^ T cells (**Figure 5a**, light blue and light green). Following intranasal administration of the protein antigen pull stimulus (‘+in’), a marked reduction in the frequency of antigen-specific CD4^+^ T cells was observed in the spleen (**Figure 5a**; dark versus light colors). For Ag85B-specific CD4^+^ T cells, two-way ANOVA even revealed a significant combined effect of αGC inclusion and the pull stimulus. Analysis of the percentage of CD69^+^CD62L^-^I-Ab tetramer^+^ (ESAT-6, 4–17 and Ag85B, 280-294) among total viable splenocytes confirmed that inclusion of αGC increased the frequency of this population, although this effect did not reach statistical significance. Consistent with the observations in C57BL6/J mice, this population was almost entirely KLRG1^-^ (data not shown). This increase was abolished in animals that received the pull stimulus, resulting in frequencies comparable to those observed in the unadjuvanted groups (**Figure 5b**).

**Figure 5:**
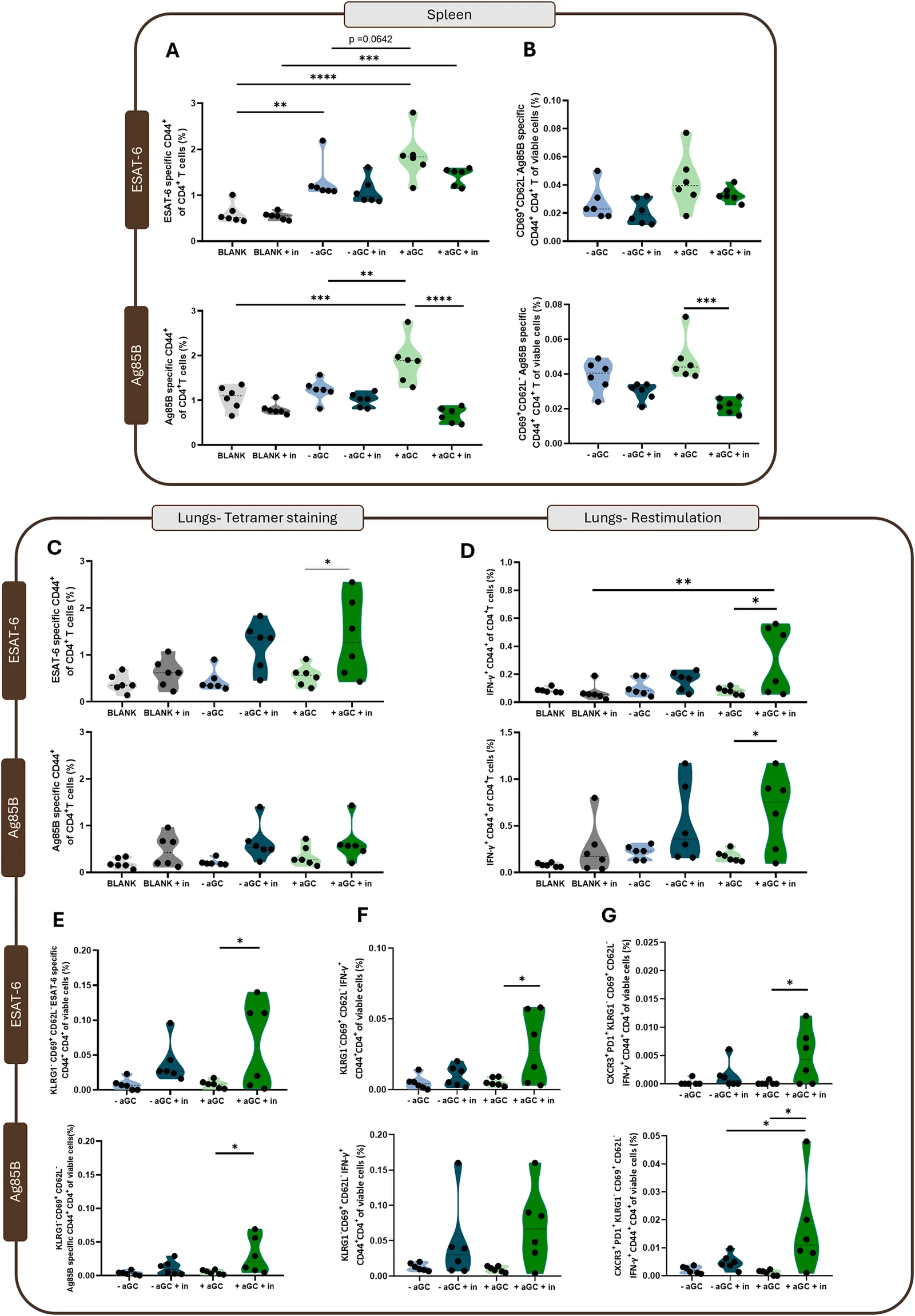
αGC inclusion contributes to the accumulation of antigen-specific T cells in the lungs with a T_RM_-associated phenotype after local TCR stimulation via local protein boosting: CB6F1 mice were immunized intramuscularly with a prime-boost dose (two-week interval) of m1Ψ-modified mRNA encoding sig-Ag85B-LAMP3 and sig-ESAT-6-LAMP3 (1:1 weight ratio) mRNA formulated in C12–200 mRNA LNPs alone (2.5 μg mRNA/mouse) or co-formulated with αGC adjuvant (n = 6) and additionally boosted at week 3 with intranasal administration of the full length proteins Ag85B and ESAT-6 (1:1 weight ratio). **(A)** Spleens were stained with I-Ab tetramers for ESAT-6 and Ag85B and **(B)** assessed for fraction of CD69^+^CD62L^-^ of viable cell population **(C)** Perfused lungs were stained with I-Ab tetramers for ESAT-6 and Ag85B and **(D)** in parallel re-stimulated with respective peptide pools, ESAT-6 and Ag85B, for 5 hours and IFN-γ production was assessed in CD4^+^ T cells. In the lungs, the frequency of CD69^+^CD62L^-^KLRG1^-^ antigen-recognizing cells was assessed in the viable cell population **(E)** after tetramer staining and **(F)** after restimulation with peptide pool. **(G)** The latter was further stained for PD1 and CXCR3, markers associated with migration and correlated with protection. Each individual point represents one animal, with lines indicating mean, n= 6 per group. P values were determined by two-way ANOVA followed by Tukey’s *post-hoc* test. The factorial design is provided in **Supplementary Table 5**, and the adjusted P values are reported in **Supplementary Table 6**. See **Supplementary Figures 3** and **5** for gating strategy.

Notably, the pull stimulus significantly increased the number of ESAT-6- and Ag85B-specific CD4^+^ T cells in the lungs, as measured by both tetramer staining (**Figure 5c**) and peptide pool restimulation (**Figure 5d**), with the highest responses observed in the αGC-adjuvanted groups. When we further examined antigen-specific T cells by assessing CD69^+^CD62L^−^KLRG1^−^ expression in the lungs, we observed similar trends, with increased frequencies following both pull-stimulus administration and αGC adjuvantation (**Figures 5e, f**). Although the decrease in splenic and increase in pulmonary antigen-specific CD4^+^ T cells were primarily driven by the pull stimulus, factorial statistical analysis revealed additional contributions from αGC adjuvantation and significant interaction effects between both, although these varied depending on the antigen and readout assessed (**Supplementary Table 5**).

TB is a pulmonary disease, and protection likely depends strongly on the presence of lung-resident memory (T_RM_) T cells and other lung-localized cell populations displaying a T_RM_ associated phenotype. Identifying such T_RM_ cells is often complex, as various cell surface markers for lung parenchymal resident memory T cells have been described before (23, 56). In this study we characterized antigen-specific lung associated CD4^+^ T cells expressing CD69, lacking CD62L and KLRG1,and co-expressing PD-1 and CXCR3 after lung perfusion. These markers have previously been associated with tissue residency-related and lung-localized T-cell phenotypes in TB vaccine studies (23, 25, 31, 57). However, in the absence of intravascular staining and additional residency markers, these cells cannot be definitively classified as bona fide lung parenchymal TRM cells. PD-1 and CD69 are used as general markers of T-cell activation while KLRG1 allows discrimination between terminally and non-terminally differentiated T cells highly relevant in context of Mtb (58). Additionally, CXCR3 was included as a chemokine receptor which is associated with rapid T-cell recruitment to infection sites such as Mtb infected lungs (23, 59). Focusing on this lung-associated T-cell phenotype, which is highly relevant to Mtb infection, we found that the effect was predominantly induced by intranasal boosting (**Figure 5g**). Nevertheless, factorial statistical analysis identified a significant interaction effect with αGC adjuvantation for Ag85B, suggesting an additional contribution of αGC to this response (**Supplementary Table 5**).

## 4 Discussion

In this study we investigated the potential of αGC-adjuvanted mRNA-LNPs as a platform for next-generation TB vaccine development. Using truncated ovalbumin as a model antigen, we first demonstrated that αGC-adjuvanted mRNA-LNPs induce robust, dose-dependent iNKT cell activation in addition to robust cellular responses. These iNKT cells may contribute directly to protection against Mtb, as they exhibit bactericidal activity that correlates with improved bacterial control and reduced bacterial burden during Mtb infection in mice (60, 61). Although elevated PD-1 expression is indicative of recent iNKT-cell stimulation, it has also been linked to the induction of hyporesponsiveness or anergy following strong αGC-mediated activation. While we previously showed that intramuscular vaccination was associated with reduced IL-10 production by iNKT cells compared to IV administration (29). Additional phenotypic and functional studies, including assessment of recall responses following secondary αGC stimulation, will be required to evaluate whether anergy contributes to the observed immune responses in this vaccination setting.

Consistent with this protective role, patients with active TB exhibit lower frequencies of iNKT-cells than individuals with latent infection, supporting their role in host defense (62, 63). Activation of iNKT by αGC has been shown to prolong survival and decrease bacterial load in Mtb-infected mice (64). These findings align with the evidence that certain mycobacterial lipids such as mycolic acid and lipoarabinomannan serve as natural CD1d ligands capable of activating iNKT cells. Indeed, vaccination with liposomes containing these natural ligands provided modest improvements in protection against Mtb infection in guinea pigs (65). Moreover, αGC-mediated iNKT activation can lead to bystander activation of other effector cells relevant to Mtb control, such as NK cells (28, 66) and gamma delta T cells (20, 48). Based on the observed dose-dependent activation of iNKT cells and cellular immunity, a 2.5 µg mRNA dose achieved an optimal balance between immunogenicity and tolerability, as reflected by minimal reactogenicity (<10% transient weight loss).

The development of effective TB vaccines traditionally focused on inducing high frequencies of T_H_1 IFN-γ producing T cells, given their well-established role in mycobacterial control (67). However, emerging evidence suggests that a broader and more multifaceted immune response is likely required to address the complex biology of Mtb (21, 68). Recent studies further indicate that immunosuppressive mechanisms within the granuloma microenvironment can contribute to poor bacterial control and disease recurrence, emphasizing the importance of strategies capable of reshaping local immune responses in addition to inducing classical T_H_1 immunity (69). A well-known example is the MVA85A vaccine candidate, which failed to confer protection in a phase 2b trial in BCG-immunized infants, despite inducing strong polyfunctional T_H_1 responses in preclinical studies (70). In line with this evolving paradigm, we explored whether the inclusion of αGC could broaden the vaccine-induced immune responses by making use of mRNA vaccines encoding ESAT-6 and Ag85B. As expected, unadjuvanted mRNA-LNPs generated a pronounced T_H_1 response, consistent with previous reports (71, 72). Upon inclusion of αGC, we observed a shift in the vaccine-induced CD4^+^ T-cell profile, characterized by the emergence of low-level T_H_2 responses and T_H_17-associated responses while maintaining substantial T_H_1 activity. This T_H_ profile aligns with the well-described response of iNKT cells upon activation with potent CD1d ligands such as αGC, characterized by early co-production of IFN-γ and IL-4. We also observed that the magnitude of these effects was antigen dependent. Beyond T_H_1 and T_H_2 CD4^+^ T-cell responses, αGC-adjuvanted vaccines induced T_H_17 CD4^+^ T cells in C57BL6/J mice. T_H_17 cells have been associated with enhanced early protection against Mtb and are often increased following mucosal immunization (73–76). Notably, a substantial fraction of these CD4^+^ T cells co-produced IFN-γ and IL-17A. This phenotype has been linked to superior bacterial control compared to IFN-γ^+^ CD4^+^ T cells (16, 77). T_H_17 cell generation is driven by cytokines such as IL-6, TGF-β and IL-23 (74). We previously observed elevated IL-6 levels in the plasma of mice vaccinated with our mRNA-LNPs, which further increased upon αGC addition (29). Polyfunctional T_H_1 CD4^+^ T cells are generally associated with effective control of TB infection (78, 79), suggesting the observed T_H_2_-_shift upon αGC addition may therefore appear disadvantageous. However, it remains unclear to what extent the other effects elicited by αGC adjuvantation will compensate for, or even outweigh, the observed reduction in this proposed correlate of protection. Future Mtb challenge studies will therefore be required to determine the overall protective efficacy of this vaccination strategy.

In contrast, experiments conducted in CB6F1 mice confirmed the immunomodulatory effects of αGC, albeit to a lesser extent given their T_H_2 biased background, and additionally revealed a CD8^+^ T response against Ag85B. The divergent effect of αGC on CD8^+^ T-cell responses to ESAT-6 and Ag85B in CB6F1/J mice is an intriguing finding that merits further investigation. Potential explanations include differences in H-2d-restricted epitope processing or MHC-I binding affinity, variations in antigen expression or stability, differences in the intrinsic immunostimulatory properties of the antigens, or competition for cross-presentation pathways. Notably, ESAT-6 has been reported to directly interact with TLR2 signaling, potentially interacting with the adjuvant-mediated effects (50). Altogether, these findings highlight the influence of host genetic background on the vaccine-induced immunity and suggest the importance of evaluating immunogenicity in diverse models, including hybrid or even humanized mice (80). Moreover, as the prime-pull experiments were only conducted in CB6F1/J mice, the extent to which the observed lung-localizing effects of αGC are conserved across genetic backgrounds remains to be determined.

As Mtb primary infects the lungs, the local induction of T_RM_ cells and T cells with T_RM_-associated characteristics is considered a critical element of protective immunity. Owing to their rapid and robust expansion upon pathogen encounter, T_RM_ cell-function as a first of line defense against Mtb infection in both mice and non-human primates (NHPs) (22, 81, 82). Confirmed by our results, conventional mRNA-LNPs are limited in their capacity to induce cells displaying a T_RM_-like phenotype in lungs (16, 72, 83). In contrast, we found that αGC incorporation substantially increased the numbers of splenic CD44^+^ CD69^+^CD62L^-^CD4^+^ T cells in both C57BL/6 and CB6F1 mice, consistent with previous reports in malaria models reported by *Ganley* et al. for CD8^+^T cells. Interestingly, in that study, IL-4 production by iNKT cells was identified as a key driver of this effect (31, 82).

Since our adjuvanted mRNA vaccine induced a population of CD44^+^ CD69^+^CD62L^-^CD4^+^ T cells in the spleen, we subsequently employed a prime-pull vaccination strategy to investigate whether mucosal antigen exposure could promote the presence of these antigen-specific T cells in the lung (24, 27, 84). Indeed, intranasal protein boosting following intramuscular administration of mRNA-LNPs, resulted in increased frequencies of antigen-specific CD4^+^ T cells in the lungs. While the pull stimulus appeared to be the primary driver of this effect, αGC may have contributed by increasing the pool of vaccine-induced antigen-experienced T cells available for accumulation following mucosal antigen exposure in the lungs. A substantial fraction of this population expressed CXCR3, PD-1, and CD69, while lacking KLRG1 and CD62L expression, indicative of an activated, non-terminally differentiated phenotype with the capacity to traffic to sites of infection in the lung and likely to exert robust effector functions (58, 82, 85, 86). Notably, similar phenotypes have previously been associated with protection against Mtb infection (25, 57) and have conferred protection upon adoptive transfer into naive mice (86).

Despite the promising findings, several limitations of this study should be acknowledged. First, the study was designed as an immunogenicity assessment and did not include an Mtb challenge experiment. Although vaccination induced broad cellular immune responses and increased frequencies of memory-like and lung-homing CD4^+^ T-cell populations, it remains unknown whether these responses confer enhanced protection against infection or disease. Consequently, the protective efficacy of this vaccination strategy should be assessed in future aerosol Mtb challenge studies before conclusions regarding its overall performance can be made, ideally in a mouse model encompassing a broad antigenic repertoire that more closely reflects the human setting. Second, a more comprehensive characterization of the memory-associated phenotype would further strengthen the findings and provide insight in their durability and trafficking. Intravascular labeling combined with assessment of lung-residency markers such as CD103 and CD49a would enable discrimination between circulating and lung-resident T-cell populations, thereby providing a more accurate characterization of the phenotype and localization of vaccine-induced memory-like T cells. Consequently, the populations described in this study should be interpreted as T cells exhibiting phenotypic characteristics associated with tissue residency rather than as bona fide T_RM_ cells. Furthermore, the CD8^+^ T-cell responses evaluated in this study were restricted to antigens encoded in the mRNA. Future studies incorporating additional antigens containing more immunodominant CD8 epitopes would be valuable to determine whether similar trends can also be observed within the CD8^+^ T-cell compartment.

Third, all experiments were performed in female mice, precluding the assessment of potential sex-dependent differences in vaccine-induced immunity. This should be taken into consideration when interpreting the results, as sex-specific differences in TB vaccine responses have previously been reported (87). Finally, the prime-pull vaccination strategy was only evaluated in CB6F1/J mice. Therefore, potential strain-dependent differences in the generation and accumulation of memory-like T-cell populations in the lungs could not be assessed.

In conclusion, our findings demonstrate that αGC can reprogram mRNA-LNP induced immunity by altering the T_H_1/T_H_2 response, promoting T_H_17-mediated immunity and facilitating the accumulation of T cells displaying phenotypic characteristics associated with tissue residency in the lung. These findings are closely aligned with immune mechanisms that have been associated with protection against Mtb. Using the Mtb antigens ESAT-6 and Ag85B, we demonstrated the induction of broad cellular immune responses, supporting the further evaluation of this vaccine platform in protective challenge studies.

## Data Availability

The raw data supporting the conclusions of this article will be made available by the authors, without undue reservation.
