# Supplementary material for: Alpha-galactosylceramide as mRNA vaccine adjuvant modulates cellular immunity against tuberculosis and contributes to antigen-specific lung-associated T cells with a TRM-associated phenotype following prime-pull strategy
Source: Front Immunol. 2026 Oct 5;17:1929726. doi: 10.3389/fimmu.2026.1929726 (PMC13677840; doi:10.3389/fimmu.2026.1929726)
Supplement: Supplementary file 1 [file DataSheet1.docx]

Supplementary Material

**Supplementary table 1: Physicochemical characterization of mRNA-LNPs used per dataset included in each figure.** Size (expressed as number mean), polydispersity index (PdI), zeta potential (mV) and mRNA encapsulation efficiency (EE%). *Measured in PBS

| **Fig.** | **Antigen** | **Formulation** | **Size (nm) (number mean)** | **PdI** | **Zeta potential (mV)** | **EE%** |
| --- | --- | --- | --- | --- | --- | --- |
| 1 | Truncated OVA | C12-200 + αGC | 73.58 | 0.094 | 9.55 | 100 |
| 1 | Truncated OVA | C12-200 - αGC | 73.36 | 0.071 | 10.87 | 100 |
| 2 and 3 | ESAT-6 / truncated Ag85B | C12-200 + αGC | 85.74* | 0.275* | -0.236 | 100 |
| 2 and 3 | ESAT-6 / truncated Ag85B | C12-200 - αGC | 71.22* | 0.225* | 0.736 | 97.62 |
| 6 | ESAT-6 / truncated Ag85B | C12-200 + αGC | 82.06 | 0.132 | -12.07 | 85.91 |
| 6 | ESAT-6 / truncated Ag85B | C12-200 - αGC | 89.75 | 0.163 | -14.00 | 92.01 |

**Supplementary table 2: Statistics on normalized body weight after administration of various doses of mRNA-LNP with and without αGC at 24, 48 and 72 hours post prime injection**. 2-way-ANOVA was performed between different doses within same vaccine formulation and vaccine formulation within dose.

| 24h - αGC | 0.5 µg | 1 µg | 2.5 µg | 5 µg |  | 48h - αGC | 0.5 µg | 1 µg | 2.5 µg | 5 µg |  | 72h - αGC | 0.5 µg | 1 µg | 2.5 µg | 5 µg |
| --- | --- | --- | --- | --- | --- | --- | --- | --- | --- | --- | --- | --- | --- | --- | --- | --- |
| 0.5 µg | \ | ns | * | **** |  | 0.5 µg | \ | * | **** | **** |  | 0.5 µg | \ | ns | ns | **** |
| 1 µg | \ | \ | ns | ns |  | 1 µg | \ | \ | * | **** |  | 1 µg | \ | \ | ns | **** |
| 2.5 µg | \ | \ | \ | ns |  | 2.5 µg | \ | \ | \ | *** |  | 2.5 µg | \ | \ | \ | **** |
| 5 µg | \ | \ | \ | \ |  | 5 µg | \ | \ | \ | \ |  | 5 µg | \ | \ | \ | \ |
|  |  |  |  |  |  |  |  |  |  |  |  |  |  |  |  |  |
| 24h + αGC | 0.5 µg | 1 µg | 2.5 µg | 5 µg |  | 48h + αGC | 0.5 µg | 1 µg | 2.5 µg | 5 µg |  | 72h + αGC | 0.5 µg | 1 µg | 2.5 µg | 5 µg |
| 0.5 µg | \ | *** | **** | **** |  | 0.5 µg | \ | ** | **** | **** |  | 0.5 µg | \ | *** | *** | **** |
| 1 µg | \ | \ | ns | ** |  | 1 µg | \ | \ | ** | **** |  | 1 µg | \ | \ | ns | **** |
| 2.5 µg | \ | \ | \ | ns |  | 2.5 µg | \ | \ | \ | **** |  | 2.5 µg | \ | \ | \ | **** |
| 5 µg | \ | \ | \ | \ |  | 5 µg | \ | \ | \ | \ |  | 5 µg | \ | \ | \ | \ |

| 24h | 0.5 µg | 1 µg | 2.5 µg | 5 µg |  | 48h | 0.5 µg | 1 µg | 2.5 µg | 5 µg |  | 72h | 0.5 µg | 1 µg | 2.5 µg | 5 µg |
| --- | --- | --- | --- | --- | --- | --- | --- | --- | --- | --- | --- | --- | --- | --- | --- | --- |
| 0.5 µg | ns | \ | \ | \ |  | 0.5 µg | ns | \ | \ | \ |  | 0.5 µg | ns | \ | \ | \ |
| 1 µg | \ | ns | \ | \ |  | 1 µg | \ | ns | \ | \ |  | 1 µg | \ | ns | \ | \ |
| 2.5 µg | \ | \ | ns | \ |  | 2.5 µg | \ | \ | ns | \ |  | 2.5 µg | \ | \ | ns | \ |
| 5 µg | \ | \ | \ | ns |  | 5 µg | \ | \ | \ | ns |  | 5 µg | \ | \ | \ | 0.0549 |

**Supplementary table 3: Nucleotide sequences of mRNA constructs used:** OVA, ESAT-6 and truncated Ag85B flanked by trafficking signals: SP LAMP-1 and LAMP-3.

| **Construct name** | **Nucleotide sequence** |
| --- | --- |
| LAMP1 SP- Truncated OVA-TM LAMP3 | atggctgctcctggatctgctagaaggcctctgctgctgcttctgttgctcctgctgctgggcctgatgcattgtgccagcgccgccccaTTTGCCAGTGGGACAATGAGCATGTTGGTGCTGTTGCCTGATGAAGTCTCAGGCCTTGAGCAGCTTGAGAGTATAATCAACTTTGAAAAACTGACTGAATGGACCAGTTCTAATGTTATGGAAGAGAGGAAGATCAAAGTGTACTTACCTCGCATGAAGATGGAGGAAAAATACAACCTCACATCTGTCTTAATGGCTATGGGCATTACTGACGTGTTTAGtTCTTCAGCCAATCTGTCTGGCATCTCCTCAGCAGAGAGCCTGAAGATATCTCAAGCTGTCCATGCAGCACATGCAGAAATCAATGAAGCAGGCAGAGAGGTGGTAGGGTCAGCAGAGGCTGGAGTGGATGCTGCAAGCGTCTCTGAAGAATTTAGGGCTGACCATCCATTCCTCTTCTGTATCAAGCACATCGCAACCAACGCCGTTCTCTTCTTTGGCAGATGTGTTTCCCCgaattctagtgaccctagcagcgactacaccatcgtgctgcctgtgatcggcgccatcgttgtgggactgtgtctgatgggcatgggcgtgtacaagatccggctgagatgccagtctagcggctaccagagaatc |
| LAMP1 SP -ESAT-6- TM LAMP3 | ATGGCAGCACCAGGTTCAGCTCGACGACCACTTTTGCTCCTCTTGCTGCTCTTGCTGCTGGGCTTGATGCACTGCGCCAGTGCTGCTACTGAACAACAATGGAACTTTGCCGGGATTGAGGCTGCCGCATCTGCCATTCAGGGCAATGTCACCAGTATTCATAGTCTCCTGGACGAAGGGAAACAATCCCTCACTAAGCTCGCTGCAGCATGGGGCGGCTCAGGAAGTGAGGCTTATCAGGGGGTTCAACAGAAATGGGACGCAACAGCCACAGAACTGAATAACGCACTTCAAAATCTTGCCCGCACCATCAGCGAAGCTGGGCAAGCAATGGCTAGTACCGAAGGCAACGTGACAGGGATGTTCGCACCTAGTTCTGACTACACAATTGTACTGCCCGTGATTGGCGCAATTGTAGTAGGGCTGTGCTTGATGGGCATGGGAGTTTATAAAATACGCCTGCGCTGCCAGTCAAGCGGTTACCAGCGAATC |
| LAMP1 SP - Truncated Ag85B- TM LAMP3 | ATGGCAGCACCAGGTTCAGCTCGACGACCACTTTTGCTCCTCTTGCTGCTCTTGCTGCTGGGCTTGATGCACTGCGCCAGTGCTGCTTTCTCTCGCCCCGGTCTTCCAGTTGAATACCTCCAAGTCCCCTCACCATCAATGGGTAGAGATATCAAGGTGCAGTTTCAGTCAGGGGGGAATAACAGTCCCGCAGTCTATCTTCTGGATGGTCTTAGGGCACAGGATGACTACAATGGGTGGGATATTAATACTCCAGCCTTTGAGTGGTATTATCAGTCCGGCCTGAGCATAGTAATGCCAGTAGGCGGCCAGAGTTCATTCTATAGTGACTGGTACAGCCCTGCCTGTGGTAAAGCCGGCTGCCAAACTTACAAATGGGAGACATTCTTGACATCTGAGTTGCCTCAATGGCTCTCCGCAAATCGAGCTGTGAAACCAACCGGCAGCGCCGCCATTGGTCTCTCAATGGCTGGTTCTTCAGCAATGATTCTTGCAGCTTACCACCCTCAGCAATTCATTTACGCTGGCTCTTTGTCTGCCCTTCTTGACCCTTCTCAGGGGATGGGGCCTAGCTTGATAGGATTGGCCATGGGGGACGCCGGAGGGTACAAAGCTGCAGATATGTGGGGTCCCAGTTCCGACCCTGCATGGGAGCGGAATGATCCCACTCAACAGATCCCTAAGTTGGTCGCTAATAACACACGCCTGTGGGTATATTGTGGCAACGGAACTCCAAACGAGTTGGGAGGTGCAAACATCCCAGCCGAATTTCTGGAGAACTTTGTACGAAGTTCCAATCTCAAATTTCAAGATGCTTATAATGCTGCAGGCGGCCACAATGCAGTTTTTAATTTTCCCCCAAATGGCACTCACAGTTGGGAGTATTGGGGAGCACAACTGAACGCCATGAAGGGTGACCTCCAGTCCTCTCTTGGGGCCGGACCTAGTTCTGACTACACAATTGTACTGCCCGTGATTGGCGCAATTGTAGTAGGGCTGTGCTTGATGGGCATGGGAGTTTATAAAATACGCCTGCGCTGCCAGTCAAGCGGTTACCAGCGAATC |

**Supplementary table 4: Antibodies, dyes and tetramers used for flow cytometry**

| Antibodies/dyes/ tetramers | Fluorophore | Clone | Dilution | Cat nr. |
| --- | --- | --- | --- | --- |
| Fixable viability dye zombie aqua (Invitrogen) | Aqua | NA | 1/400 | L34957 |
| Fixable viability dye zombie aqua (Biolegend) | Aqua | NA | 1/400 | 423102 |
| Fc Block TruStain FcX (CD16/32) | NA | 93 | 1/200 | 101320 |
| H-2Kb/SIINFEKL tetramer  (OVA tetramer) | BV421 | NA | 1/100 | NA |
| I-A/ QQWNFAGIEAAASA (  ESAT-6 tetramer) | PE | NA | 1/50 | NA |
| I-A/ FQDAYNAAGGHNAVF  (Ag85B tetramer) | BV421 | NA | 1/50 | NA |
| mCD1d PBS-57 tetramer  (iNKT-cell tetramer) | PE | NA | 1/100 | NA |
| TCRβ | APC Efluor780 | H57-597 | 1/100 | 47-5961-82 |
| PD-1 | FITC | MH5A | 1/100 | 143705 |
| CD8a | APC Fire750 | 53-6.7 | 1/100 | 100765 |
| TCRβ | APC | H57-597 | 1/100 | 109212 |
| CD4 | APC Fire 750 | rm4-5 | 1/100 | 116020 |
| CD44 | PerCP | IM7 | 1/100 | 103035 |
| KLRG1 | BV605 | 2F1/KLRG1 | 1/100 | 138419 |
| CD69 | FITC | H12F3 | 1/100 | 104505 |
| CD62L | PE Vio770 | MEL14-H2.100 | 1/100 | 130-119-124 |
| CD4 | FITC | rm4-5 | 1/100 | 100510 |
| CD8a | APC | 53-6.7 | 1/100 | 100712 |
| CD4 | BV650 | rm4-5 | 1/100 | 100547 |
| PD1 | APC Fire 750 | H12F3 | 1/100 | 135239 |
| CXCR3 | BV421 | CXCR3-173 | 1/50 | 126521 |
| IFNγ | PE | XMG1.2 | 1/50 | 505808 |
| TNFα | BV421 | MP6XT22 | 1/50 | 506327 |
| IL4 | BV605 | 11B11 | 1/50 | 504125 |
| IL17A | PerCP | TC11-18H10.1 | 1/50 | 506943 |
| IL2 | APC | JES6-5H4 | 1/50 | 503809 |

**Supplementary figure 1: Gating strategy panel used in figure 1 to analyze iNKT cells** (illustrated for 0,5 µg C12-200 mRNA-LNP with αGC included in the formulation).

**Supplementary figure 2: Gating strategy panel of used in figure 1 to analyze OVA-specific CD8^+^ T cells** (illustrated for 5 µg C12-200 mRNA-LNP).

**Supplementary figure 3: Gating strategy of panel used in figure 2, 4 and 5** (illustrated for gating of splenocytes of C57BL6 after vaccination with C12-200 mRNA-LNP adjuvanted with αGC).

**Supplementary figure 4: Gating strategy of panel used in figure 3 and 4** (illustrated for gating of splenocytes of C57BL6 after vaccination with C12-200 mRNA-LNP adjuvanted with αGC).

**Supplementary figure 5: Gating strategy of panel used in figure 5** (illustrated for gating of lung cells of CB6F1 after vaccination with C12-200 mRNA-LNP adjuvanted with αGC and intranasally boosted with proteins).

**Supplementary figure 6: % KLRG1^-^ CD44^+^CD69^+^CD62L^-^CD4^+^T ESAT-6 and Ag85B specific CD4^+^T cells after vaccination with mRNA-LNPs encoding TB antigens ESAT-6 and Ag85B in C57BL/6 mice**. Female CB6F1 mice were administered 2.5 µg mRNA-LNPs with or without the adjuvant αGC encoding full length Mtb antigens ESAT-6 and Ag85B coupled with sig-lamp signaling elements (1:1 weight ratio) during a prime boost injection with 2 weeks interval. Using additional surface staining, % of KLRG1**^-^** of CD44^+^CD69^+^CD62L^-^CD4^+^T cells were determined using ESAT-6 and Ag85B-specific I-Ab tetramers in spleen (**A,B** respectively). P values were determined one-way ANOVA followed by Tukey’s post-hoc test.

**Supplementary figure 7: T_H_17 cells after vaccination with mRNA-LNPs encoding TB antigens ESAT-6 and Ag85B in C57BL/6 mice**. Female CB6F1 mice were administered 2.5 µg mRNA-LNPs with or without the adjuvant αGC encoding full length Mtb antigens ESAT-6 and Ag85B coupled with sig-lamp signaling elements (1:1 weight ratio) during a prime boost injection with 2 weeks interval. After re-stimulation of splenocytes with ESAT-6 **(A)** and Ag85B **(B)** separately cytokine producing T_H_17 CD4^+^ T cells were analyzed by either IL-17^+^ or IFN-γ^+^ IL-17^+^ production. Boolean gating was applied to analyze the co-expression of the polyfunctional cytokine producing CD4^+^ T cells. P values were determined two-way ANOVA followed by Tukey’s post-hoc test.

**Supplementary table 5. Full two-way factorial ANOVA analysis of Figure 5.** Statistical analysis was performed using a two-way ANOVA with αGC (present/absent) and intranasal pull stimulus (present/absent) as fixed factors. Reported values correspond to the main effect of αGC, the main effect of pull, and the interaction term (αGC × pull). All groups consisted of n=6 animals. Reported p-values correspond to the omnibus F-tests from the two-way ANOVA and are therefore not pairwise-comparison adjusted. Adjusted Tukey post-hoc p-values for pairwise comparisons are provided in **Supplementary Table 6.**

| **Figure** | **Endpoint** | **αGC effect** | **Pull effect** | **interaction** | **N** |
| --- | --- | --- | --- | --- | --- |
| A | ESAT-6 specific CD44^+^ of CD4^+^ T cells (%)- spleen | **P < 0.0001** | **P = 0.0380** | P = 0.3516 | 6 |
| A | Ag85B specific CD44^+^ of CD4^+^ T cells (%)-spleen | **P = 0.0195** | **P < 0.0001** | **P = 0.0001** | **6** |
| B | CD69^+^CD62L^-^ ESAT-6 specific  CD44^+^ CD4^+^ T of viable cells (%)- spleen | **P = 0.0192** | P = 0.1503 | P = 0.8583 | 6 |
| B | CD69^+^CD62L^-^ Ag85B specific  CD44^+^ CD4^+^ T of viable cells (%) -spleen | P = 0.8488 | **P < 0.0001** | **P = 0.0207** | **6** |
| C | ESAT-6 specific CD44^+^ of CD4^+^ T cells (%)- lungs | **P = 0.0487** | **P = 0.0003** | P = 0.2108 | 6 |
| C | Ag85B specific CD44^+^ of CD4^+^ T cells (%)-lungs | P = 0.3968 | **P = 0.0023** | P = 0.7706 | 6 |
| D | ESAT6- IFN-γ^+^ re-stimulation lungs | **P = 0.0448** | **P = 0.0218** | **P = 0.0281** | **6** |
| D | Ag85B- IFN-γ^+^ re-stimulation lungs | P = 0.0741 | **P = 0.0013** | P = 0.3911 | 6 |
| E | KLRG1^-^ CD69^+^ CD62L^-^ ESAT-6 specific  CD44^+^ CD4^+^ of viable cells (%) | P = 0.3599 | **P = 0.0055** | P = 0.3796 | 6 |
| E | KLRG1^-^ CD69^+^ CD62L^-^  Ag85B specific CD44^+^ CD4^+^ of viable cells (%) | P = 0.1166 | **P = 0.0130** | P = 0.1847 | 6 |
| F | KLRG1^-^CD69^+^ CD62L^-^ IFN-γ^+^  CD44^+^CD4^+^ of viable cells (%)- ESAT-6 | P = 0.0764 | **P = 0.0150** | P = 0.0910 | 6 |
| F | KLRG1^-^CD69^+^ CD62L^-^ IFN-γ^+^  CD44^+^CD4^+^ of viable cells (%)- Ag85B | P = 0.4851 | **P = 0.0080** | P = 0.4585 | 6 |
| G | CXCR3^+^ PD1^+^ KLRG1^-^ CD69^+^ CD62L^-^  IFN-γ^+^ CD44^+^ CD4^+^of viable cells (%)-ESAT-6 | P = 0.1578 | **P = 0.0151** | P = 0.1357 | 6 |
| G | CXCR3^+^ PD1^+^ KLRG1^-^ CD69^+^ CD62L^-^  IFN-γ^+^ CD44^+^ CD4^+^of viable cells (%)- Ag85B | P = 0.0615 | **P = 0.0190** | **P = 0.0463** | **6** |

**Supplementary Table 6. Significant Tukey-adjusted pairwise comparisons for Figure 5 endpoints.** Adjusted p-values from Tukey's multiple-comparison test are shown for statistically significant pairwise comparisons (p < 0.05) among experimental groups included in Figure 5.

| **Figure** | **Endpoint** | **Comparison** | **Tukey-adjuested p-value** |
| --- | --- | --- | --- |
| A | ESAT-6 specific CD44^+^ of CD4^+^ T cells (%)- spleen | BLANK vs LNP+αGC | <0.0001 |
|  |  | BLANK vs LNP-αGC | 0.0089 |
|  |  | BLANK vs LNP +αGC + IN | 0.0019 |
|  |  | LNP +αGC vs BLANK + IN | <0.0001 |
|  |  | LNP +αGC vs LNP -αGC + IN | 0.0037 |
|  |  | LNP-αGC vs BLANK + IN | 0.0045 |
|  |  | BLANK + IN vs LNP +αGC + IN | 0.0009 |
| A | Ag85B specific CD44^+^ of CD4^+^ T cells (%)-spleen | BLANK vs LNP+αGC | 0.0002 |
|  |  | LNP+αGC vs LNP−αGC | 0.0042 |
|  |  | LNP+αGC vs BLANK+IN | <0.0001 |
|  |  | LNP+αGC vs LNP+αGC+IN | <0.0001 |
|  |  | LNP+αGC vs LNP−αGC+IN | <0.0001 |
|  |  | LNP−αGC vs LNP+αGC+IN | 0.0177 |
| B | CD69^+^CD62L^-^ ESAT-6 specific  CD44^+^ CD4^+^ T of viable cells (%)- spleen | LNP+αGC vs LNP−αGC+IN | 0.0442 |
| B | CD69^+^CD62L^-^ Ag85B specific  CD44^+^ CD4^+^ T of viable cells (%) -spleen | LNP+αGC vs LNP+αGC+IN | 0.0002 |
|  |  | LNP+αGC vs LNP−αGC+IN | 0.0063 |
|  |  | LNP−αGC vs LNP+αGC+IN | 0.0116 |
| C | ESAT-6 specific CD44^+^ of CD4^+^ T e cells (%)- lungs | LNP vs LNP+αGC+IN | 0.0142 |
|  |  | LNP+αGC vs LNP+αGC+IN | 0.0363 |
|  |  | BLANK vs LNP+IN | 0.0363 |
|  |  | BLANK vs LNP+αGC +IN | 0.0080 |
| D | ESAT6- IFN-γ^+^ re-stimulation lungs | LNP vs LNP+αGC+IN | 0.0350 |
|  |  | LNP+αGC vs LNP+αGC+IN | 0.0116 |
|  |  | BLANK vs LNP+αGC +IN | 0.0140 |
|  |  | BLANK + IN vs LNP+αGC +IN | 0.0082 |
| D | Ag85B- IFN-γ^+^ re-stimulation lungs | LNP+αGC vs LNP+αGC+IN | 0.0466 |
|  |  | BLANK vs LNP+αGC +IN | 0.0109 |
| E | KLRG1^-^ CD69^+^ CD62L^-^ ESAT-6 specific  CD44^+^ CD4^+^ of viable cells (%) | LNP vs LNP+αGC+IN | 0.0437 |
|  |  | LNP+αGC vs LNP+αGC+IN | 0.0462 |
| E | KLRG1^-^ CD69^+^ CD62L^-^  Ag85B specific CD44^+^ CD4^+^ of viable cells (%) | LNP vs LNP+αGC+IN | 0,0273 |
|  |  | LNP+αGC vs LNP+αGC+IN | 0,0406 |
|  |  | LNP vs LNP+αGC+IN | 0.0214 |
|  |  | LNP+αGC vs LNP+αGC+IN | 0.0246 |
